# How the geometry of cities determines urban scaling laws

**DOI:** 10.1098/rsif.2020.0705

**Published:** 2021-03-17

**Authors:** Carlos Molinero, Stefan Thurner

**Affiliations:** ^1^ Complexity Science Hub Vienna, Josefstädterstrasse 39, 1080 Vienna, Austria; ^2^ Austrian Institute of Technology, Giefinggasse 2, 1210 Vienna, Austria; ^3^ CASA, University College London, 90 Tottenham Court Road, London W1T 4TJ, UK; ^4^ Section for the Science of Complex Systems, CeMSIIS, Medical University of Vienna, Spitalgasse 23, 1090 Vienna, Austria; ^5^ Santa Fe Institute, 1399 Hyde Park Road, Santa Fe, NM 87501, USA

**Keywords:** fractal geometry, urban scaling, spatial networks, population size, agglomeration effects

## Abstract

Urban scaling laws relate socio-economic, behavioural and physical variables to the population size of cities. They allow for a new paradigm of city planning and for an understanding of urban resilience and economics. The emergence of these power-law relations is still unclear. Improving our understanding of their origin will help us to better apply them in practical applications and further research their properties. In this work, we derive the basic exponents for spatially distributed variables from fundamental fractal geometric relations in cities. Sub-linear scaling arises as the ratio of the fractal dimension of the road network and of the distribution of the population embedded in three dimensions. Super-linear scaling emerges from human interactions that are constrained by the geometry of a city. We demonstrate the validity of the framework with data from 4750 European cities. We make several testable predictions, including the relation of average height of cities and population size, and the existence of a critical density above which growth changes from horizontal densification to three-dimensional growth.

## 1. Introduction

One of the surprising findings in urban science is that many of the hundreds of quantities and variables that characterize the dynamics, functioning, and performance of a city exhibit power law relations. These are called *scaling laws*, meaning that a quantity X depends on a variable p (such as population) in a power-law fashion. In particular, this means that *X* is related to the population of the city as

1.1 $$X\propto p^{\gamma },$$

where *γ* is the scaling exponent and *p* represents population size. Several quantities scale linearly (*γ* = 1) with population, such as water consumption, housing, or the number of employments [1]. However, non-trivial urban scaling laws abound and appear in a vast number of different contexts. For example, scaling laws with respect to population size were found for GDP [2,3], the number of patents [4], walking speed [5] or crime rates [2]. The associated scaling exponent for these relations appears to be in a range of *γ* ∼ 1.1–1.2 and are said to scale super-linearly with the population. The fact that different variables share the same exponent (belong to the same class) points to a potential shared underlying cause for the emergence of those values. For other quantities, the total length of the road network [6], the length of electrical cables [1], the number of facility locations [7] or of petrol stations [8], the associated scaling exponent is often found in a range of *γ* ∼ 0.8–0.9.^1^ This is referred to as sub-linear scaling.

Note that technically it is all but trivial to quantify urban scaling exponents reliably and consistently and some works question the measurement techniques used in a large fraction of the literature [9,10]. A major difficulty is a proper definition of city boundaries, which is at the heart of some of the discrepancies in several works [11–13]. Depending on the notion of city boundaries, it has been shown that exponents for a system can vary substantially, sometimes even from a sub-linear to a super-linear behaviour. To avoid these issues, we propose an approach to obtain city boundaries directly from population data; for details, see electronic supplementary material, section S2.

Urban scaling is of immediate relevance for a number of reasons. First, they allow us to compute the detailed economies of scale for a single city. They relate the size of cities to efficiency gains or losses for a wide range of quantities that determine life in cities. For example, if a quantity like the total length of the road network scales sub-linearly with population size, this means that the cost per person decreases with city size; the larger a city becomes the more efficient it will be with respect to this variable. If the population of city A is *x* times larger than B, sub-linear scaling means that the *per capita* effort in city A is a factor *x*^*γ*−1^ < 1 less than in B. Second, they allow us to rescale variables establishing a correct comparison between different cities. If one would directly compare, for example, the *per capita* GDP of a large and a small city, due to the presence of super-linear scaling, the large city will have a bias toward larger GDP values that is only due to scaling, and not to, for example, better management. Third, since urban scaling laws appear to be largely similar across countries and cultures, they can be used for urban planning, in particular for anticipating consequences of rapid growth. If a city is expected to double in size within the next decades, depending on the scaling exponents, dozens of performance indicators, growth rates, infrastructure costs, etc. can be inferred and used proactively in city planning. Given a level of growth, scaling laws pose clear constraints to urban performance indicators and possibilities for change. It is therefore of fundamental importance to understand the nature of these scaling laws and to give a clear and concise reasoning for the emergence of the specific values of their exponents.

Until today, a general understanding of urban scaling laws is still under debate. In particular, the origin of the values of the exponents, why they cluster in specific ranges, and why the super-linear and the sub-linear exponent tend to add up to two [14] call for a coherent and comprehensive explanation.

*Scaling laws* are present in almost any field of science. They appear in second-order phase transitions, where at the critical point, universality classes have been defined for different systems that share the same set of critical exponents [15–17]. Scaling laws are embedded in Newtonian physics, where the inverse quadratic law arises from the dimension of the spherical area around the mass. Allometric relations are found across all biological species (Kleiber’s Law [18]) as a result of fractal geometry [19]. Earthquake magnitudes follow power-law distributions [20]. Power laws appear in preferential attachment processes, in the distribution of the degrees of networks [21] and a countless number of other examples [22].

In the context of urban environments, various explanations have been given for the emergence of scaling laws. Some use underlying network structures of the social tissue. In [23] the authors focus on the social network structure of cities understood as a hierarchical tree. This allows them to define a distance in the tree that is used to calculate the probability of people interacting. This is then used to calculate the overall productivity of the city as proportional to the number of interactions. They are able to reproduce the super-linear exponent of interactions; however, the approach uses several assumptions that cannot be tested, such as the tree-like structure of the social ties or the decay of interactions with the distance in that topological structure. In [24] the authors propose a geographical network embedded in a fractal Euclidean space, to explain the sub- and super-linear exponents. However, they need to create two parameters that are difficult to measure, complicating the model. Another approach is based on a path-dependent evolution of innovations [25], where cumulative cycles of innovation give rise to the growth of cities, that further lead to the next round of innovations. The authors present a longitudinal explanation of scaling exponents that depend on the cycle of innovation of each sector, relegating more mature technologies to smaller cities, while new products are introduced in the largest cities. This explanation of economic innovation cycles does not explain, however, other scaling exponents that relate to physical quantities, such as the scaling of infrastructure, or the number of gas stations.

Two recent works propose to explain the observed exponents partly on the basis of the underlying geometrical structure of cities. In [26] the authors consider growth models for cities in which an equilibrium between costs and benefits produces the scaling exponents. They assume that cities are space-filling fractals. Most cities have a fractal dimension of less than 2 since in every settlement there exist empty spaces and voids of different sizes, such as parks and open public spaces, which lead to fractal dimensions that fall consistently in a range between 1.2 and 1.93, depending on the city [27–31]. The model of [14] builds on the notion that interactions between people decay with distance in a specific way, and assumes that the fractal dimension of the population, *d*_*p*_, is equal to that of the infrastructure. In particular, they expect it to be around *d*_*p*_ ∼ 1.7. However, given that the population lives in three-dimensional buildings, its fractal dimension should be expected to be definitively larger than that of the road network, i.e. larger than 2. Both models use geometric arguments, but do not attempt to directly relate the observed scaling exponents to the geometry of a city.

This is exactly what we propose in this work. Scaling laws can often be explained directly from the geometry of the underlying structures of a system. Classic examples include Galileo’s understanding of the relation between the shape of animals and their body mass [32], the understanding of the allometric scaling laws in biology on the basis of the fractal geometry of the branching of vascular systems [19,33,34], or the scaling laws of river basins given by their fractal geometry [35]. In the same spirit, we provide a simple and a direct geometrical explanation of urban scaling exponents, derived from the fractal geometry of cities. Cities across countries, latitudes and cultures are different—and so is their geometry. How should cities that are significantly different in their geometry lead to similar scaling exponents? To answer this question we focus on the ratio of two geometric aspects of a city, the fractal dimension of its infrastructure (street networks) [28,36–38], and the fractal dimension of the population, meaning the dimension of the object that represents the spatial distribution of the population in a city. The fractal of the population can be imagined as the cloud of people that is obtained by identifying the position of every person in three dimensions.

The present study aims to show how the values of the exponents of a subset of scaling variables arise from the fractal geometry of a city, and therefore directly explain the particular scaling. The approach presented in this paper is applicable to the study the scaling of spatially distributed variables with respect to population size. The sub-linear exponent of planar infrastructure (roads, pipes, gas stations, etc.) is exemplified by the *total length of roads* throughout the text. The super-linear exponent arises from the *number of social interactions* in a population and all variables that are proportional to it (criminality, GDP, etc.).

The basic idea of this work is very general and simple: any scaling exponent for a spatially distributed variable can be understood as the ratio of the fractal dimension of a measured object (such as the total length of road networks) and the fractal dimension of the population.

Most infrastructure exists along road networks, gas/water/electricity lines follow the pattern of streets, so as to remain accessible. Also, gas stations are located along streets. These infrastructure networks will therefore expose the same (or a very similar) fractal dimension. This means that they share the same scaling exponent, which allows us to talk more generally about the sub-linear scaling class (*γ*_sub_). Other variables that do not have a spatial component fall outside the logic of this work. Variables with a spatial component with a different fractal dimension can still be explained with the logic presented in this paper, but will lead to different values of scaling exponents. An example of these is how the area of a city scales with its population, which will be its fractal dimension, 2, divided by the fractal dimension of the population, *d*_*p*_.

Within this geometric framework, we are not only able to understand the origin of specific super- and sub-linear scaling exponents in a new light and why they add up to 2, we can also predict a number of geometric scaling laws, such as the average height of a city, the length of the road network, the area that contains a city, and the number of interactions. All these predictions are confirmed empirically to a large level of precision.

In the remainder of the paper, we will use the notation ∝, ∼ , = to mean proportionality, similarity and strict equality, respectively (see electronic supplementary material, section S8).

## 2. Methods

**Figure 1.**
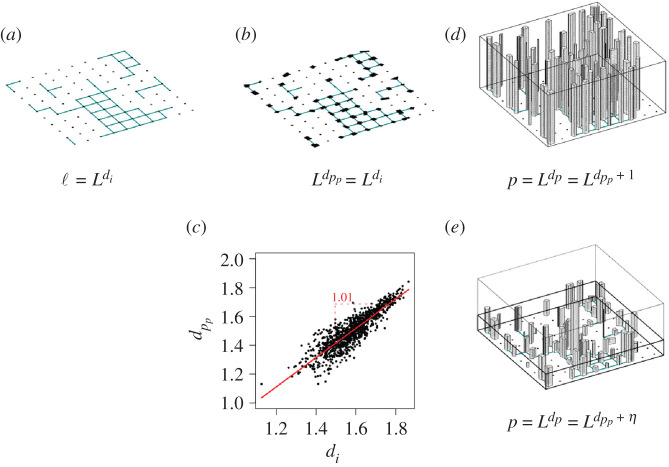
(*a*) Street network in a section of the city of size *L*. The length of the street network with fractal dimension *d*_*i*_ expands with the linear scale *L* as $\ell \propto L^{d_{i}}$. (*b*) Buildings are located along the street network and are attached to it. Since people live and work mostly in buildings, the fractal dimension of the ‘projected population’ (the actual population fractal projected onto the two-dimensional surface, where streets are embedded) should have a similar fractal dimension $d_{\;p_{\;p}}=d_{i}$. This is shown in (*c*), where both dimensions show a strong linear correlation for every city in the UK, with a slope of 1. (*d*) If all buildings were to have the same height, the fractal dimension of the population, *d*_*p*_, should be the projected population dimension plus 1, $d_{\;p}=d_{\;p_{\;p}}+1$. (*e*) More realistically, since not all buildings have the same height, the fractal dimension of the populations is $d_{\;p}=d_{\;p_{\;p}}+\eta$, where *η* captures the fractal dimension along the third dimension.

The physical aspect of a city is largely determined by its buildings and its street network. Street networks can be characterized with a fractal dimension 1 < *d*_*i*_ < 2, as measured in the literature [27–31]. Given this exponent, the average length of roads that is contained in a square of *ε* × *ε* is ${\left\langle \ell \right\rangle }_{\epsilon }={\left\langle \ell \right\rangle }_{\epsilon_{0}}{\left(\epsilon /\epsilon_{0}\right)}^{d_{i}}$, where *ε*_0_ is the minimum length scale that represents the lowest resolution limit for the measurement of the fractal dimension, ${\left\langle \ell \right\rangle }_{\epsilon_{0}}$ is a city specific constant that is equal to the average length of roads in a square of side *ε*_0_, and the total length of the street network, ℓ, can be approximated when *ε* approaches *L*, the linear extension (scale) of the city, as schematically shown in figure 1*a*. Therefore, ℓ can be expressed as

2.1 $$\ell ∼{\left\langle \ell \right\rangle }_{\epsilon_{0}}{\left(\frac{L}{\epsilon_{0}}\right)}^{d_{i}},$$

where ${\left(L/\epsilon_{0}\right)}^{d_{i}}$ is the number of boxes of size *ε*_0_ within a square of side *L*. Note that this is formula states that the total length of roads is the average length of roads inside a square, multiplied by the number of non-empty squares. This is true regardless of whether the road network is a fractal (1 < *d*_*i*_ < 2), or whether the city would occupy the entire surface, *d*_*i*_ = 2. Since *L*/*ε*_0_ is dimensionless, ℓ is given in the units in which we measured ${\left\langle \ell \right\rangle }_{\epsilon_{0}}$, in our case metres.

The population is distributed along buildings, which are located on the fractal generated by the streets. Since houses extend into the third dimension, the population is distributed as a fractal in space, with dimension *d*_*p*_, where *d*_*i*_ ≤ *d*_*p*_ ≤ *d*_*i*_ + 1. Given a three-dimensional grid of cubes of linear size *ε*, the average number of people living in an *ε*-box is ${\left\langle p\right\rangle }_{\epsilon }={\left\langle p\right\rangle }_{\epsilon_{0}}{\left(\epsilon /\epsilon_{0}\right)}^{d_{\;p}}$, where ${\left\langle p\right\rangle }_{\epsilon_{0}}$ is a city-specific constant, the number of people living in a box of size *ε*_0_. It is related to the number of people per square metre that can live in a flat. If we now choose a box size that contains the entire city (*ε* ∼ *L*), the population can be expressed as

2.2 $$p∼{\left\langle p\right\rangle }_{\epsilon_{0}}{\left(\frac{L}{\epsilon_{0}}\right)}^{d_{\;p}},$$

where *L* is the side of the box that contains the city, ${\left(L/\epsilon_{0}\right)}^{d_{\;p}}$ is the number of non-empty boxes contained in a square of side *L* and ${\left\langle p\right\rangle }_{\epsilon_{0}}$ is the average number of people inside a box of side *ε*_0_. To verify how well equations (2.1) and (2.2) are realized empirically, consult electronic supplementary material, figure S5. Note that we view the population distributed in space as a cloud of points, where every person is represented as a point, and its location is given by the three-dimensional coordinates of the apartment where the person lives.

Unfortunately, three-dimensional data of the population do not exist. What we can obtain is a planar, projected version onto the surface of the city that can be expressed as a fractal. The average number of people that are projected into an *ε* × *ε* square is ${\left\langle p_{\;p}\right\rangle }_{\epsilon }={\left\langle p_{\;p}\right\rangle }_{\epsilon_{0}}{\left(\epsilon /\epsilon_{0}\right)}^{d_{\;p_{\;p}}}$, where ${\left\langle p_{\;p}\right\rangle }_{\epsilon_{0}}$ is the average number of projected population into a square of size *ε*_0_. Note that ${\left\langle p_{\;p}\right\rangle }_{\epsilon_{0}}$ is not independent of the size of the city, since it will absorb the dimensionality reduction that occurred when we projected the population from three-dimensional to its two-dimensional representation. Writing the whole population as a function of its two-dimensional projected version for *ε* ∼ *L*, we get

2.3 $$p∼{\left\langle p_{\;p}\right\rangle }_{\epsilon_{0}}{\left(\frac{L}{\epsilon_{0}}\right)}^{d_{\;p_{\;p}}}.$$

Both planar fractal dimensions *d*_*i*_ and $d_{\;p_{\;p}}$ can be directly measured from their respective datasets [39,40] using box-counting (see electronic supplementary material, section S3, for a detailed explanation), and, as shown in figure 1*c*, *d*_*i*_ and $d_{\;p_{\;p}}$ show very closely related values. This happens because people live in buildings, and buildings are aligned along streets, therefore, we expect $d_{\;p_{\;p}}$ to be close to *d*_*i*_.

The estimation of the dimension of the three-dimensional population fractal, *d*_*p*_, is harder to obtain due to limitations in the data. The three-dimensional information of the population distribution is not directly available. To compute it nevertheless, we propose to decompose $d_{\;p}=d_{\;p_{\;p}}+\eta$ into its planar (or projected) part, $d_{\;p_{\;p}}$, and a component that captures the ‘fractality’ of the vertical component, *η*, which can be approximated from data on the height of buildings [39] (figure 1*e*).

Open Street Maps [39] is digitalizing three-dimensional information of cities. It is a work in progress and some countries (such as the UK) are more complete than others. For each city, we obtain the average number of levels in a building, 〈*h*〉, and the maximum number of levels, *h*_*m*_, as well as how many buildings were digitized in that city. Given these data, we obtain the average population per level in an *ε* × *ε* square, ${\left\langle p_{h}\right\rangle }_{\epsilon }={\left\langle p_{\;p}\right\rangle }_{\epsilon }/\langle h\rangle$.

To compute *d*_*p*_ with box-counting, we need the average number of people in three-dimensional boxes of different sizes *ε*. Technically, box-counting needs at least two different box sizes, which is, of course, an extremely poor approximation. However, from the data we only know the average population per box at two specific *ε* values in a reliable way. Assuming that the typical floor is 3 m high, then for *ε* = 3 m, one box fits into every level and the population in each box will be the population per level in a square of the same size, ${\left\langle p\right\rangle }_{3}={\left\langle p_{h}\right\rangle }_{3}={\left\langle p_{\;p}\right\rangle }_{\epsilon_{0}}/\left\langle h\right\rangle {\left(3/\epsilon_{0}\right)}^{d_{\;p_{\;p}}}$. The second box size is the maximum height of the city, *ε* = 3*h*_m_. The population in each box will be equal to its projected version, ${\left\langle p\right\rangle }_{3h_{m}}={\left\langle p_{\;p}\right\rangle }_{3h_{m}}={\left\langle p_{\;p}\right\rangle }_{\epsilon_{0}}{\left(3h_{m}/\epsilon_{0}\right)}^{d_{\;p_{\;p}}}$. With these two values, we can approximate

2.4 $$d_{\;p}∼\frac{\log{\left\langle p\right\rangle }_{3h_{m}}-\log{\left\langle p\right\rangle }_{3}}{\log3h_{m}-\log3}=d_{\;p_{\;p}}+\frac{\log\langle h\rangle }{\log h_{m}}\;$$

The fractal dimension of the population is the fractal dimension of the planar projection, plus the fractal dimension of the vertical component and *η* = log〈*h*〉/log *h*_m_.

To obtain the sub-linear exponent we combine equations (2.1) and (2.2), to express ℓ in terms of the population allowing us to derive the sub-linear exponent, $\ell \propto p^{d_{i}/d_{\;p}}$. We denote the sub-linear scaling exponent by *γ*_sub_ = *d*_*i*_/*d*_*p*_. Note that we would not be able to use equations (2.1) and (2.3) to obtain the same derivation, since as will be shown in the following, ${\left\langle p_{\;p}\right\rangle }_{\epsilon_{0}}$ depends on the size of the city.

The number of social interactions (approximated by the number of cellphone calls) as a function of city size follows a scaling law with a super-linear scaling exponent that is close to 1.12 [41]. We can calculate how this exponent emerges in our framework as a consequence of the geometry of the street network and how people are distributed in three dimensions.

Using equations (2.2) and (2.3), we can write

2.5 $${\left\langle p_{\;p}\right\rangle }_{\epsilon_{0}}∼{\left\langle p\right\rangle }_{\epsilon_{0}}{\left(\frac{L}{\epsilon_{0}}\right)}^{dp-d_{\;p_{\;p}}}.$$

Interactions occur when people leave their apartments and wander into the streets, go to a bar, or to the local supermarket. Therefore, the number of interactions is controlled in a rough approximation by the projected version of the population onto the streets. If we have ${\left\langle p_{\;p}\right\rangle }_{\epsilon_{0}}$ people in a square of size *ε*_0_, the maximal number of their interactions is ${\left\langle p_{\;p}\right\rangle }_{\epsilon_{0}}{\left({\left\langle p_{\;p}\right\rangle }_{\epsilon_{0}}-1)∼\langle p_{\;p}\right\rangle }_{\epsilon_{0}}^{2}$. The instantaneous number of interactions, *N*, in a city is proportional to that value (times the probability that a potential link becomes an actual interaction), multiplied by the number of locations in which that can happen, which is ${\left(L/\epsilon_{0}\right)}^{d_{p_{p}}}$. Therefore, using equations (2.5) and (2.2) we get

2.6 $$N\propto {\left\langle p_{\;p}\right\rangle }_{\epsilon_{0}}^{2}{\left(\frac{L}{\epsilon_{0}}\right)}^{d_{\;p_{\;p}}}\propto {\left(\frac{L}{\epsilon_{0}}\right)}^{2d_{\;p}-d_{\;p_{\;p}}}∼p^{2-\frac{d_{\;p_{\;p}}}{d_{\;p}}}∼p^{2-\gamma_{\mathrm{sub}}}.$$

Here we used the empirical finding that the fractal dimension of the projected population follows the dimension of the street network, $d_{\;p_{\;p}}∼d_{i}$ (figure 1*c*). We identify the scaling exponent obtained from the interaction densities as the super-linear exponent, *γ*_sup_ = 2 − *γ*_sub_. The addition rule that states that the two exponents add up to 2 follows from this derivation.

We can further derive the exponent for the projected average population, which comes simply from writing equation (2.5) as a function of the population, ${\left\langle p_{\;p}\right\rangle }_{\epsilon_{0}}\propto {\left(L/\epsilon_{0}\right)}^{d_{\;p}-d_{\;p_{\;p}}}∼p^{1-d_{\;p_{\;p}}/d_{\;p}}∼p^{1-\gamma_{\mathrm{sub}}}$ (using equation (2.2)). Since ${\left\langle p_{\;p}\right\rangle }_{\epsilon }={\left\langle p_{\;p}\right\rangle }_{\epsilon_{0}}{\left(\epsilon /\epsilon_{0}\right)}^{d_{\;p_{\;p}}}$ and ${\left(\epsilon /\epsilon_{0}\right)}^{d_{\;p_{\;p}}}$ is approximately independent of the population, we have that also ${\left\langle p_{\;p}\right\rangle }_{\epsilon }\propto p^{1-\gamma_{\mathrm{sub}}}$. Moreover, since by definition, ${\left\langle p_{h}\right\rangle }_{\epsilon }={\left\langle p_{\;p}\right\rangle }_{\epsilon }/\langle h\rangle$, we naturally have that $\langle h\rangle {\left\langle p_{h}\right\rangle }_{\epsilon }\propto p^{1-\gamma_{\mathrm{sub}}}$. The full picture presents itself when we understand that growing vertically requires more effort than increasing density, and consequently, as long as one can increase ${\left\langle p_{h}\right\rangle }_{\epsilon }$, then 〈*h*〉 will remain constant and ${\left\langle p_{h}\right\rangle }_{\epsilon }\propto p^{1-\gamma_{\mathrm{sub}}}$. At some point, the density of people per level in a square will saturate and will become a constant, forcing 〈*h*〉 to absorb all the growth and thus $\langle h\rangle \propto p^{1-\gamma_{\mathrm{sub}}}$.

## 3. Results

**Figure 2.**
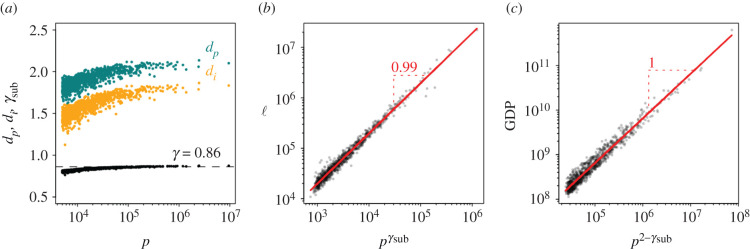
(*a*) Fractal dimensions of the street network, *d*_*i*_, (orange) and for the population, *d*_*p*_, (blue) for 1000 cities in the UK as a function of their population size *p*. While the fractal dimensions are strongly size dependent, their ratio, *γ*_sub_ = *d*_*i*_/*d*_*p*_ (black), is not. It is found to be approximately constant, *γ*_sub_ ∼ 0.86. (*b*) The sub-linear relation between street length ℓ and $p^{\gamma_{\mathrm{sub}}}$ is shown for the empirical data. It follows the theoretical prediction almost perfectly (red line). (*c*) As an example for a super-linear scaling law the relation between city GDP and $p^{2-\gamma_{\mathrm{sub}}}$ is shown. Red lines represent the linear regression and every dot is a city.

We show in figure 2*a* the measured dimensions *d*_*i*_ and *d*_*p*_ for 1000 UK cities as a function of their population. Results for other major European countries (DE, FR, ES, IT) can be found in electronic supplementary material, figure S5(a). Both dimensions exhibit a clear dependence on population size, *p*, therefore their ratio shows a small dependence on city size which saturates as cities grow larger, as explained in depth in the electronic supplementary material, section S1. This means that the scaling exponent cannot be characterized by a single value which needs to be considered in order to avoid contradictions. Figure 2*b* portrays the sub-linear scaling exponent, *γ*_sub_, which as shown, allows us to almost perfectly reproduce the empirical length of street networks ℓ.

It has been argued [26] that the observed super-linear scaling exponents of several variables (GDP, criminality) can be explained as a consequence of the super-linear scaling exponent of social interactions in cities. This assumption means that we can use a measurable indicator such as the GDP to validate the result obtained from our framework (number of interactions). We show the GDP [42] for UK cities in comparison to our theoretical prediction of the number of interactions in figure 2*c*. It behaves as $p^{2-\gamma_{\mathrm{sub}}}$, as predicted.

**Figure 3.**
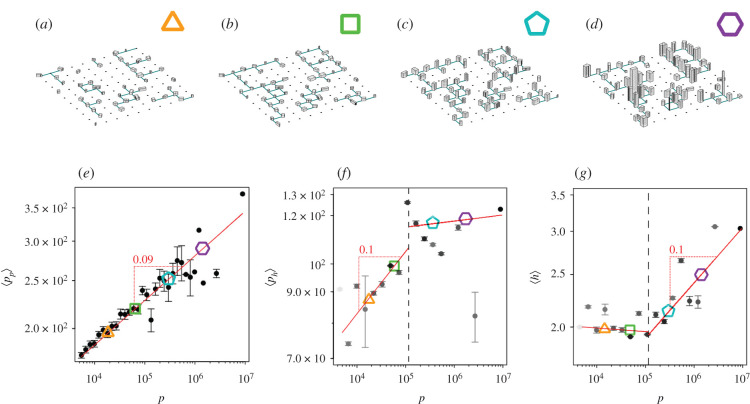
Schematic of how a city grows. (*a*,*b*) For low populations, the city expands and densifies mostly horizontally, buildings have one or a few levels. (*c*,*d*) From a critical population size upward, buildings begin to grow into the third dimension. In this regime, the urban scaling law in the average building heights, 〈*h*〉, is expected to hold. (*e*) The average value of the projected version of the population grows with the same exponent 1 − *γ*_sub_ showing an approximated slope of 0.09. Obviously, there is no critical size, as expected. (*f* ) At the critical population level, the average value of the population in each floor, as measured by 〈*p*_*h*_〉, saturates, and can no longer grow. Up to this point, the city densifies to absorb the increase of the population. (*g*) The scaling behaviour of the average building height of UK cities, 〈*h*〉, is clearly scaling, and follows the theoretical prediction for the exponent 1 − *γ*_sub_. Scaling only appears for populations larger than 100 000, above the saturation level of 〈*p*_*h*_〉. The red line indicates the scaling region with a slope of 0.10. Below the critical population the growth is marginal. In (*e*), (*f* ) and (*g*) each point is the average for similar sized cities using log-bins. In the case of (*f* ) and (*g*), this is a weighted average, using as weights the number of buildings digitized in each city.

In figure 3, we show the growth of the average number of levels 〈*h*〉, density of people in a square ${\left\langle p_{p}\right\rangle }_{\epsilon }$, and people per level ${\left\langle p_{h}\right\rangle }_{\epsilon }$, and compare them to our theoretical predictions. In a low-density population regime, the growth of projected population ${\left\langle p_{\;p}\right\rangle }_{\epsilon }$ is absorbed by an increase in the density of people per level ${\left\langle p_{h}\right\rangle }_{\epsilon }$, and as density reaches saturation and becomes constant, the number of levels of the city 〈*h*〉 starts to increase to allow for further growth. We can see in figure 3*e* that ${\left\langle p_{\;p}\right\rangle }_{\epsilon }$ maintains a power-law relation with respect to population growth to absorb the dimensionality reduction from representing the three-dimensional population with a two-dimensional dataset, which makes it scale with an exponent 1 − *γ*_sub_. We observe that for population sizes below 10^5^ people practically no growth in the average number of levels is observed; the city grows horizontally, by a densification process in the two-dimensional plane with an exponent of 1 − *γ*_sub_ (figure 3*f*). Above 10^5^ people, the saturation of density of people per level is reached and cities begin to grow into the third dimension, with a scaling exponent of 1 − *γ*_sub_, as predicted (figure 3*g*). A different approach to study scaling of heights was presented in [43].

We summarize the urban scaling exponents that are explainable within the proposed geometric framework in table 1. Since the exponent varies with city size, we used the results for the largest cities. For the UK, we have the values *γ*_sub_ ∼ 0.86 and *d*_*p*_ ∼ 2.14.

**Table 1.** Urban scaling exponents explainable with our geometric framework. The measured values are obtained from the UK. The maximum values of *γ*_sub_ obtained for the different countries are $\gamma_{\mathrm{sub}}^{\mathrm{UK}}=0.86$, $\gamma_{\mathrm{sub}}^{\mathrm{FR}}=0.79$, $\gamma_{\mathrm{sub}}^{\mathrm{DE}}=0.81$, $\gamma_{\mathrm{sub}}^{\mathrm{ES}}=0.82$, $\gamma_{\mathrm{sub}}^{\mathrm{IT}}=0.81$.

quantity	theory	measured	reference
street length, ℓ	*γ*_sub_	0.86	here
average height, 〈*h*〉	1 − *γ*_sub_	0.10	here, figure 3*e*
interactions, *N*	2 − *γ*_sub_	1.12	[41]
city GDP	2 − *γ*_sub_	1.12	[44]
proj. pop., 〈*p*_*p*_〉	1 − *γ*_sub_	0.09	here, figure 3*f*
city area	$\frac{2}{d_{\;p}}$	0.91	here, electronic supplementary material, figure S10

## 4. Discussion

Urban scaling laws are deeply related with the ways humans move, live, act and interact within a city. The way these actions happen is strongly governed and constrained by its specific geometry. A geometrical measure that is able to capture these constraints is the ratio between the fractal dimension of the infrastructure (street) network and the fractal that represents how the population is distributed in three-dimensional space. We claim that some urban scaling laws emerge as a result of the interplay between the structures where people are located and the structures they can move on. This is the reason why the scaling exponents can be expressed in terms of this ratio. We explicitly showed that this geometric framework leads to predictions that are in excellent agreement with actual data for the scaling laws of the length of street networks and heights of buildings. For the latter, the value of the exponent is determined by how the heights of buildings change, once cities start to expand into the third dimension, which happens at a critical city density when the population approaches approximately 100 000 people. This change of regime is probably related to the critical population that determines the transition from a mono-centric to a poly-centric city [45,46]. The existence of two different scaling regimes has been shown previously in [47]. Further, the geometric ratio explains a number of very different aspects of scaling in a perfectly coherent way.

In summary, a fractal geometry perspective on cities allows us to accomplish the comprehensive understanding of the origin of the sub-linear exponent associated to infrastructure networks and the super-linear scaling exponent of social interactions on the basis of geometry alone, the relationship between both, and finally, to systematically relate the fractal dimensions of geometric objects to the exponents of the observed scaling laws. With the latter we predict several scaling relations and verified their existence with data. The geometrical perspective has also allowed us to calculate for the first time individual exponents for each city which shows that the exponent depends on city size.

Cities exhibit surprisingly stable geometrical ratios across countries and cultures, showing even a similar dependence to population size, and have been called universal [48]. The extent of this universality is currently under debate. Other studies have shown that exponents depend on the way cities are defined [12] and that measures of the same variable differ, depending on the number of samples used [11], as we explain in the electronic supplementary material, section S1. The nature of this behaviour is still unknown and we will explore it deeper in a future global scale study. We believe that one of the main drivers behind the universal exponents might be that cities occupy space in similar ways, producing objects of similar ratios of fractal dimensions which drive the related scaling exponents to a very specific range.

## Endnote

1 In [7] the authors find an exponent of 0.66, however, with respect to another variable. This variable (density) approximately scales with 0.15 with respect to population, so that effectively they have an exponent of 0.15 + 0.66 = 0.81.

## Data accessibility

All data sources used in this paper belong to the category of third-party open data and can be freely accessed under the umbrella of the corresponding institutions as referenced in the text. These datasets are: Global human settlement layer (GHS) created by the European Commission and accessible at http://ghsl.jrc.ec.europa.eu/ghs_pop.php; OpenStreetMaps data accessible at https://planet.osm.org and Data on the GDP of cities from Eurostat accessible at https://ec.europa.eu/eurostat/web/rural-development/data.
